# Structural investigations of La_0.6_Sr_0.4_FeO_3−*δ*_ under reducing conditions: kinetic and thermodynamic limitations for phase transformations and iron exsolution phenomena†

**DOI:** 10.1039/c7ra12309d

**Published:** 2018-01-15

**Authors:** Thomas Götsch, Lukas Schlicker, Maged F. Bekheet, Andrew Doran, Matthias Grünbacher, Corsin Praty, Mizuki Tada, Hirosuke Matsui, Nozomu Ishiguro, Aleksander Gurlo, Bernhard Klötzer, Simon Penner

**Affiliations:** Institute of Physical Chemistry, University of Innsbruck A-6020 Innsbruck Austria simon.penner@uibk.ac.at +43 512 507 58003; Fachgebiet Keramische Werkstoffe, Institut für Werkstoffwissenschaften und technologien, Technical University Berlin 10623 Berlin Germany; Advanced Light Source, Lawrence Berkeley National Laboratory Berkeley California 94720 USA; Department of Chemistry, Graduate School of Science, Nagoya University Furo-cho, Chikusa-ku Nagoya 464-8601 Japan; RIKEN SPring-8 Center Sayo Hyogo 679-5148 Japan

## Abstract

The crystal structure changes and iron exsolution behavior of a series of oxygen-deficient lanthanum strontium ferrite (La_0.6_Sr_0.4_FeO_3−*δ*_, LSF) samples under various inert and reducing conditions up to a maximum temperature of 873 K have been investigated to understand the role of oxygen and iron deficiencies in both processes. Iron exsolution occurs in reductive environments at higher temperatures, leading to the formation of Fe rods or particles at the surface. Utilizing multiple *ex situ* and *in situ* methods (*in situ* X-ray diffraction (XRD), *in situ* thermogravimetric analysis (TGA), and scanning X-ray absorption near-edge spectroscopy (XANES)), the thermodynamic and kinetic limitations are accordingly assessed. Prior to the iron exsolution, the perovskite undergoes a nonlinear shift of the diffraction peaks to smaller 2*θ* angles, which can be attributed to a rhombohedral-to-cubic (*R*3̄*c* to *Pm*3̄*m*) structural transition. In reducing atmospheres, the cubic structure is stabilized upon cooling to room temperature, whereas the transition is suppressed under oxidizing conditions. This suggests that an accumulation of oxygen vacancies in the lattice stabilize the cubic phase. The exsolution itself is shown to exhibit a diffusion-limited Avrami-like behavior, where the transport of iron to the Fe-depleted surface-near region is the rate-limiting step.

## Abstract 2

A dependence of structural transformation and iron exsolution on chemical environment and reducing conditions is proven for the perovskite La_0.6_Sr_0.4_FeO_3−*δ*_.

## Graphical abstract

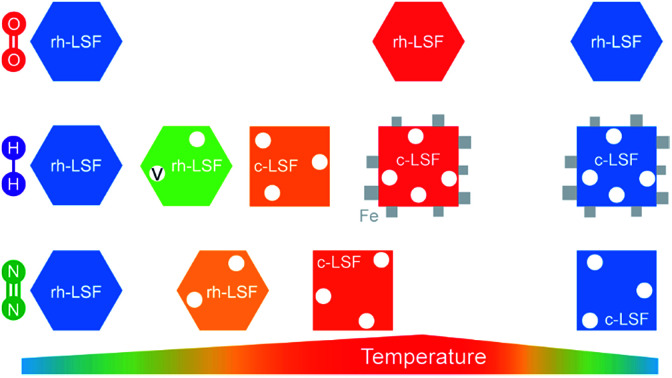

## 1. Introduction

Solid oxide fuel cells (SOFCs) usually consist of a YSZ electrolyte and additionally, a Ni/YSZ cermet anode. As cathode material, a lanthanum strontium manganite (LSM) perovskite is employed frequently.^1^ To account for the drawbacks of fuel cell operation, also all-perovskite SOFCs are proposed, lowering the operating temperatures and improving the chemical and physical compatibilities of the different fuel cell parts,^2^ as well as aiding reversible SOFC/solid oxide electrolysis cell (SOEC) operation,^3^ by making use of the versatile properties of these so-called mixed ionic and electronic conductor materials (MIECs). This duality of conductivity properties renders perovskites suitable as electrode as well as electrolyte materials.

Lanthanum strontium ferrite (LSF, La_0.6_Sr_0.4_FeO_3−*δ*_) is one of the most employed model systems, which has also been proposed as a new SOFC anode component, and therefore has been subject to numerous electrocatalytic activity studies,^3–5^ including also employing B-site doping.^6–8^ A previous study revealed that LSF exhibits iron exsolution under reducing conditions using hydrogen as reduction agent at higher temperatures, resulting in the formation of metallic Fe rods on the surface.^9,10^ Segregated iron also affects SOEC operation, causing a pronounced enhancement of the water-splitting kinetics in H_2_/H_2_O upon cathodic polarization.^11^ Despite being obviously beneficial for the latter purpose, the exsolution process and as such, the exposed iron surface is strongly dependent on the experimental parameters (*e.g.* temperature, heating rate, dry/wet conditions) and, thus, notoriously difficult to control. The latter renders use of the material without proper knowledge of its thermodynamic and kinetic limitations very difficult. The resulting structural instability would essentially prevent this material from being used as SOFC anodes, where the exposure to reducing conditions and the volume change of the unit cell may cause structural breakdown of the cell. Recent work also suggested that the exsolved iron morphology can be steered by proper adjustment of the experimental parameters, in particular the reduction potential of the reducing agent.^10^ Exsolution, which also occurs in pure iron oxide materials, and as such is of paramount technological importance, *e.g.* in highly reducing atmospheres in blast furnaces for iron production from raw iron ores, is strongly dependent on a delicate balance of iron transport from the bulk to the surface and the imminent reduction of the latter at the surface (and the formation and stability of the associated oxygen vacancies).^12^ Upon reduction control, *i.e.* if the formation of oxygen vacancies at the surface is the rate-limiting step, iron rod and whisker formation is observed, whereas if iron (cation) transport through the bulk to the surface is rate-limiting, several iron nuclei at the surface are generated, eventually leading to a dense percolated iron layer at the surface. Exsolution phenomena are not limited to LSF, but are in fact a common phenomenon of many other perovskite systems exhibiting elements capable of forming stable low-valent ionic species.^13,14^ A recent publication also highlighted how defined metal/perovskite systems could also be generated upon exsolution by both controlling the intrinsic material (*e.g.* doping level or level of non-stoichiometry) and extrinsic experimental properties (*e.g.* gas environment, oxygen partial pressure).^15^ Thus, it is of general importance to characterize the iron exsolution in detail in order to eventually be able to control it under SOFC operation and at the same time to understand the thermodynamic and kinetic prerequisites of the preceding steps. The latter are of special importance also for perovskite systems, since these are known to give rise to pronounced phase transformations as a function of gas atmosphere at temperatures before eventual exsolution phenomena set in ref. 16. Control and proper assessment of the structural transformations prior to eventual exsolution are therefore imperative.

To achieve this goal, we employ a number of *ex situ* and especially also *in situ* techniques, including X-ray photoelectron spectroscopy (XPS), *in situ* X-ray diffractometry (XRD), *in situ* thermogravimetric analysis coupled with differential scanning calorimetry and mass spectrometry (TGA-DSC-MS) and scanning X-ray absorption near edge spectroscopy (XANES) to investigate the behavior of LSF under inert and reducing conditions.

## 2. Experimental

### 2.1. Sample preparation

La_0.6_Sr_0.4_FeO_3−*δ*_ powders (obtained from Sigma Aldrich) were used for all studies. For the *ex situ* measurements, the samples were treated in flowing (1 mL s^−1^), dry hydrogen (with the water removed using a liquid nitrogen cooling trap) up to selected temperatures.

### 2.2. *Ex situ* characterization

X-ray photoelectron spectra were recorded using a Thermo Scientific MultiLab 2000 spectrometer, equipped with an Alpha 110 hemispherical sector analyzer and monochromated Al Kα radiation. A flood gun provides electrons with a kinetic energy of 6 eV for charge compensation of the samples. As charge reference, a small amount of graphite was added to the surface of the powders. The asymmetric C 1s peak was subsequently shifted to 284.2 eV. For the determination of the oxidation states of Fe, the Fe 2p peaks of both FeO and Fe_2_O_3_ were recorded separately as reference spectra and fitted to the respective sample spectra. In order to obtain a spectrum of pure FeO (being prone to disproportionation into Fe and Fe_3_O_4_), iron(ii) oxalate (FeC_2_O_4_) was thermally decomposed using the *in situ* heating stage within the spectrometer chamber by heating it to a temperature of 573 K. Further details are given in a previous work.^17^

Transmission electron microscopy images were collected by a 200 kV JEOL analytical high-resolution electron microscope equipped with an energy-dispersive X-ray spectrometer (EDX) and an electron energy-loss spectrometer (EELS).

Spatially-resolved Fe K-edge XANES spectra of the samples were recorded at the Spring8 synchrotron facility. For the experiments, the X-ray energy was scanned at 270 points in the range of 7.0 to 7.2 keV. For the Fe Kα X-ray fluorescence (XRF) mapping experiments, the focused X-ray beam (at 7.3 keV) was scanned every 150 nm in the respective *x*- and *y*-axes. The X-ray fluorescence was measured using a four-element silicon drift detector. For all measurements, LSF was dispersed in ethanol and dropped onto a SiN membrane. The LSF particle was then selected using an optical microscope. The XRF and XANES measurements were conducted under He flow to ensure inert conditions.

### 2.3. *In situ* characterization

*In situ* X-ray diffraction was performed at beamline 12.2.2 at the Advanced Light Source using a beam energy of 25 keV (*λ* = 0.4959 Å). The sample holders were quartz capillaries with inner diameters of 500 μm, and the gas injection was done using a 300 μm capillary with cut-open ends.^18^ The pattern acquisition time was 20 s and the heating as well as cooling rates were 10 K min^−1^ for all experiments. A Perkin Elmer flat panel detector (XRD 1621, with dark image and strain correction) is used to record the XRD patterns every 25 seconds.

Simultaneous Thermal Analysis (STA) was conducted in an STA 409 PC LUXX (Netzsch, Germany) device under Air, He and 5% H_2_ in Ar using 100 mg of sample powder that was placed in alumina crucibles. The released gaseous species (*m*/*z* = 2, 16, 17, 18, 44) were analyzed simultaneously in an OMNi Star GSD 320 mass spectrometer (Pfeiffer Vacuum, Germany).

## 3. Results and discussion

### 3.1. Electron microscopy characterization

**Fig. 1:**
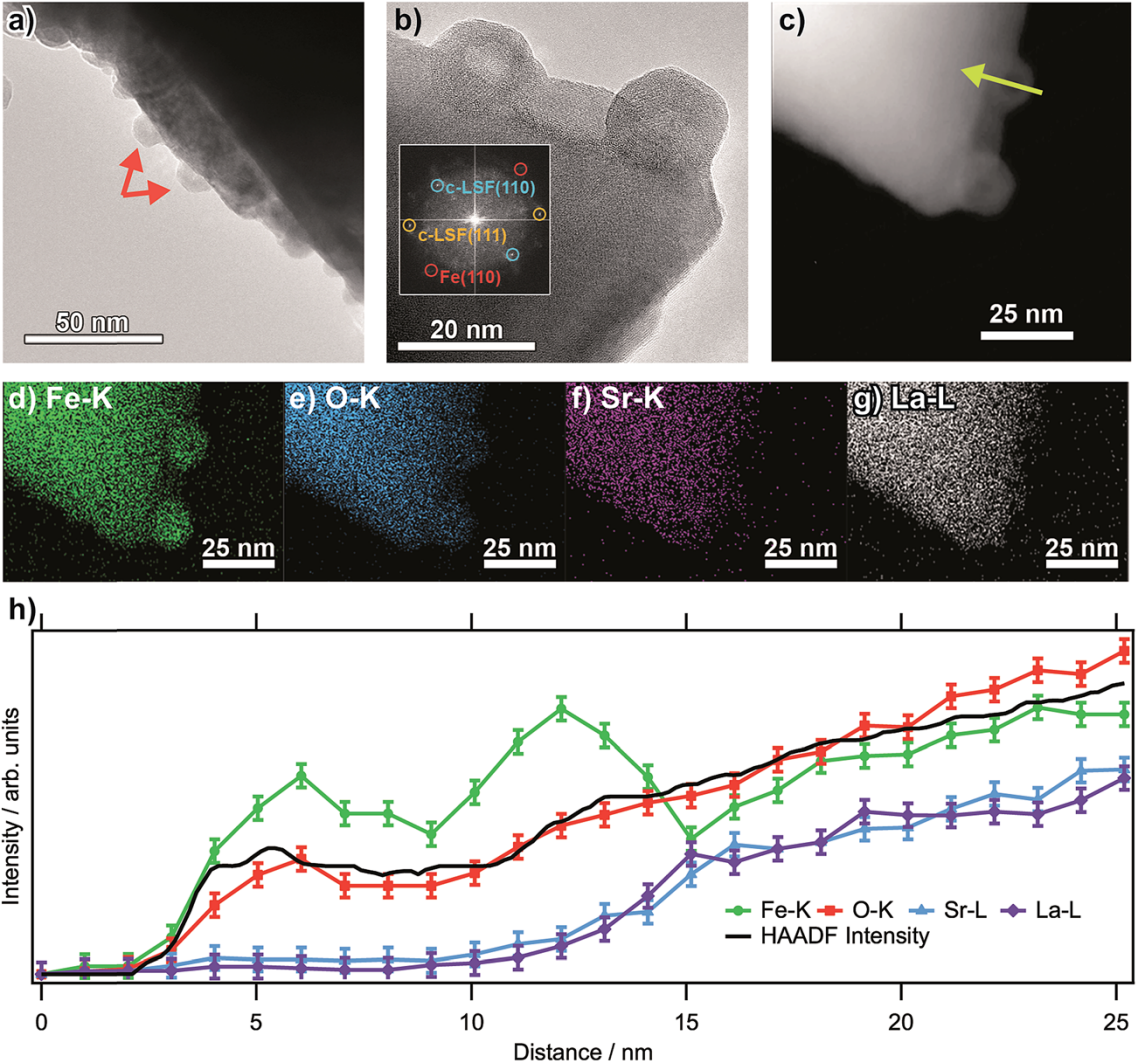
(a) Bright field image of an LSF grain after reduction in dry H_2_ at 873 K (1 h, 1 mL s^−1^), showing the exsolved Fe particles decorating the surface. A high-resolution image is shown in (b), with the respective FFT shown as inset. (c) HAADF image. (d–g) Individual EDX maps of Fe–K, O–K, Sr–K and La–L. (h) EDX line profiles across one particle along the arrow shown in the HAADF image shown in (c).

Fig. 1a shows transmission electron micrographs from an LSF sample that has been pre-reduced at 873 K. The bright field image reveals that the surface of the perovskite is decorated with small (about 10 to 15 nm in diameter, marked by arrows) particles consisting of exsolved metallic iron. These particles exhibit a hexagonal shape, as is expected of body-centered cubic structures. The high-resolution image in (b) corroborates this assignment: the fast-Fourier transform (inset) shows signals stemming both from cubic LSF, as well as from bcc iron. The latter can be shown to originate from the two particles at the surface. The high-angle annular dark field (HAADF) image in (c), essentially displaying element-specific chemical contrast (constant density and thickness provided), shows the same region as the high-resolution image. The contrast of these particles is not homogeneous within the particles, revealing a darker core and accordingly, a brighter shell. This implicates that the average atom mass of the core is lower, constant particle thickness provided. A line scan recorded along the green line depicted in the HAADF image (Fig. 1b) reveals that neither La, nor Sr are present at the location of the particle, suggesting that is an iron (oxide) species. The same can also be seen in the individual EDX maps of Fe–K, O–K, Sr–K and La–L (Fig. 1d–g). The concentration profiles of Fe and O both reveals a dip at the location of the dark contrast, indicating that the particles are hollow. This originates from the (nanoscale) Kirkendall effect during the passivation of the Fe particles upon transport in air. This well-known effect usually occurs upon reaction of a solid with its gaseous, liquid or solid surroundings and has *e.g.* been shown to take place upon oxidation of Ni and Co nanoparticles,^19^ but recently also for the reaction between metals and oxides to form intermetallic phases upon reduction.^20^ In the present case, an initial iron oxide hull is formed around the exsolved metal particles, through which diffusion occurs for the further reaction to proceed. If the diffusion of oxygen into the particles is slower than that of Fe ions outwards, the center of the particles will accordingly be depleted in Fe, while the outward growth results in a net increase of the particle diameter, leaving behind a hollow core. This translates directly into the line profile (which was taken along the line depicted in the HAADF image; the concentration profiles are in Fig. 1h).

### 3.2. *In situ* X-ray characterization

**Fig. 2:**
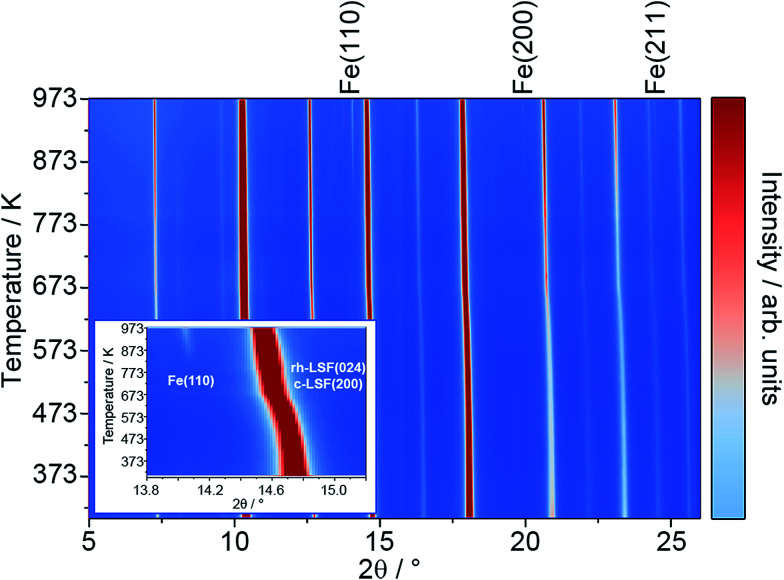
*In situ* XRD patterns (*λ* = 0.4959 Å) taken in an Ar/H_2_ mixture (5% H_2_, heating rate: 10 K min^−1^) at temperatures up to 973 K. The signals of metallic Fe are labeled above the plot. The inset highlights the region around the Fe(110) and rh-LSF (024) reflexes.

To characterize the exsolution reaction in more detail, *in situ* XRD was performed using synchrotron radiation. The results of these temperature-programmed diffraction measurements in an Ar/H_2_ mixture (5% H_2_, heating rate of 10 K min^−1^) are displayed in Fig. 2. The most prominent feature of the diffractograms, in addition to the presence of rhombohedral LSF signals (space group *R*3̄*c*) is the appearance of the Fe reflexes at around 823 K (*e.g.* the Fe(110) peak at a 2*θ* value of around 14.0°, also compare the inset). The respective Fe signals are denoted by labels above the plot.^21^

**Fig. 3:**
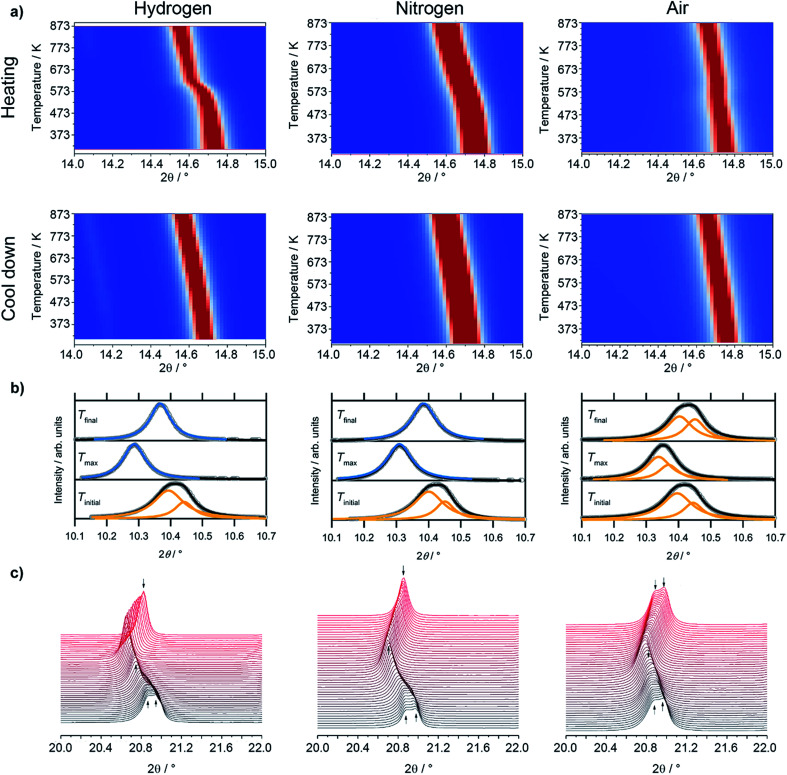
(a) Detailed view of the (024) reflex evolution of rhombohedral LSF as a function of the temperature in various gas environments (*λ* = 0.4959 Å). The top row depicts the heating period and the second one the cool down phase. In (b), fits for the (110) and (104) peaks of rhombohedral LSF, which cannot be fully resolved, and cubic c (200) reflexes are shown at room temperature in the beginning (*T*_initial_) and the end (*T*_final_) of the experiment, as well as at the maximum temperature (*T*_max_). As peak shapes, Lorentz functions were employed and a linear background was used to fit the data. (c) During heating/cooling cycles, the rh-LSF (220) and (208) reflexes are shown to coalesce into the single c-LSF (220) signal at higher temperatures for H_2_ and N_2_ environments.

Most interestingly, a pronounced kink in all LSF reflexes below 673 K can be observed. This is especially visible in the zoomed-in area in the inset. In this region, the peaks shift to smaller scattering angles, corresponding to larger unit cell dimensions. Since this could indicate a possible phase transformation prior to and leading to the Fe exsolution, this experiment was repeated with gas atmospheres of pure H_2_, inert N_2_ and air. The resulting diffractograms of the area around the most prominent (024) reflex of LSF (at 2*θ* = 14.7°) are highlighted in Fig. 3a. In these experiments, the samples were heated to maximum temperatures of 873 K (top row) and subsequently cooled down using the same rate as before (bottom row). The panels in Fig. 3b, on the other hand, show details of the (110) and (104) peaks (unresolved due to the close match of the lattice spacings) for the initial state (*i.e.* 298 K), for the maximum temperatures and again at 298 K after cooling down. In the waterfall plots in Fig. 3c, the evolution of the (220) and (208) signals for a temperature cycle (including heating and cooling) is displayed.

During the heating phase in H_2_, the LSF (024) peak exhibits the expected linear shift due to thermal expansion. However, a pronounced non-linear feature, causing a sudden kink just below 623 K, is also seen. This rapid change in the lattice dimension suggests that a phase transition takes place at this temperature. This is conceivable as substituted perovskites, as LSF, are stable in multiple crystal structures, such as hexagonal,^22^ cubic,^23^ tetragonal,^24^ or orthorhombic phases.^25^ Upon cooling, this feature is missing and the lattice only undergoes linear shrinking due to the reversal of the thermal expansion coefficient, indicating that the phase transition is not reversible. The position of the (024) peak at the end of the heating/cooling cycle interestingly does not match the initial position: it is strongly shifted to lower 2*θ* values, corroborating the irreversibility of the phase transformation under the chosen experimental reduction conditions. Shown in more detail in (b), it is revealed that the combined (110)/(104) peak at first shifts from 10.411° to 10.286° upon heating, and then relaxes to 10.368° after re-cooling to 298 K. In addition, the peak width decreases significantly upon heating: the full width at half maximum (FWHM) is 0.137° in the initial state, but decreases to 0.087° at *T*_max_ and remains at 0.080° in the final re-cooled state. This could either result from grain growth, or from a phase transformation leading to a crystal structure with only a single reflex at this position (*i.e.* the cubic perovskite, crystallizing in the space group *Pm*3̄*m*,^23^ with the (200) reflex at this scattering angle). Additionally, it possibly is an effect of an increased order inside the lattice due to the heat-treatment, or a combination of all three. The (220)/(208) reflexes displayed in Fig. 3c show a sharpening, with both peaks seemingly merging into a single one during the heating phase. This is further evidence of a phase transition, and the single peak continues to grow in intensity and remains sharp during the cooling of the sample.

Evidence of the phase transformation leading to a structure with a peak only containing a single reflex is the respective peak shape: while in the initial state a single Lorentzian function is not sufficient to describe the peak shape due to the asymmetry and the general shape of the tails of the peak, it is possible to fit a single Lorentz shape describing *e.g.* the cubic (200) reflex to the peaks at *T*_max_ and *T*_final_. These fitting procedures are shown in Fig. 3, panels (b). For the initial state, a combination of two peaks corresponding to the predicted positions of the (110) and (104) reflexes of the rhombohedral LSF polymorph lead to a satisfactory fit of the experimental data, further corroborating the phase transformation. In fact, Rietveld analysis (Fig. 6) directly confirms that the phase transformation results in the formation of the cubic polymorph. Furthermore, in the plot depicting the heating phase in H_2_, the Fe(110) peak can be observed at approximately 14.1° arising at temperatures above 723 K, which is 100 K lower than in the H_2_/Ar mixture (see Fig. 2), which can be attributed to the higher H_2_ partial pressure, resulting in an increase of the reduction potential. The Fe reflex is still present during cooling, where it shifts similarly to the LSF reflex due to the thermal contraction. If the same procedure is applied in unreactive N_2_ environments (middle panels of Fig. 3a), there, too, is a strong non-linear change in the peak position upon heating, although it is not as pronounced as for the hydrogen. The curvature of the peak position, and, thus, the phase transformation, appears to be smeared out over a broader temperature region, which can also be observed in the waterfall plot in Fig. 3c. During cooling, the same behavior is seen as in the H_2_ environment, with the lattice contracting as a result of the decrease of the temperature. The (110)/(104) LSF peak behavior (b) appears similar to hydrogen: in the initial state, the peak maximum is observed at 10.420°, shifting to 10.310° at 873 K and relaxing back to 10.385° after the cool-down. Furthermore, the peak widths are altered too: before the experiment, the apparent FWHM of the combination peak is 0.132°, shrinking to 0.097° at the maximum temperature and remaining at that value upon reaching room temperature again. The apparent difference to H_2_ is that no metallic Fe can be observed. Upon heating the sample in air (right panels in Fig. 3a), as a non-reductive reference gas environment, there is no strong change in the peak position as compared to the other gases. In fact, there is only a linear shift observed both upon heating and cooling, corroborated by the analysis of the thermal evolution of the (110)/(104) reflexes: a change from 10.415° to 10.351° is observed, but the shift at the highest temperature is not as large as before. Moreover, the cooled specimen exhibits the same peak position as before the heating cycle (10.425°). The peak shapes of all initial and final states render the description by a single function impossible, whereas the combination of two peaks as described before leads to a good superposition of the experimental data and the envelope of the two functions. The peak at 873 K in air seems to be a special case, as it is possible to sufficiently describe it by the combined (110)/(104) reflexes as well as by a single cubic (200) peak. Also, the peak shape seems to be a mixture between the initial and final states of the other two experiment runs, with the peak being sharper than at *T*_initial_, but broader than is the case for the other gas environments at *T*_max_. Furthermore, the peak width after the heating/cooling cycle is very similar to the initial states of the previous experiments, with the widths being 0.136°, 0.102° and 0.127° before the heating, at the maximum temperature and after the heating in air, respectively.

Rietveld analyses (*cf.*Fig. 6) strongly suggest that in air, the phase transformation does not take place. The initial LSF sample has a rhombohedral *R*3̄*c* crystalline structure. During heat treatment in the different atmospheres, changes in crystalline structure occur, alongside segregation of iron in case of treatment in hydrogen. To evaluate the changes in crystalline structure, the *in situ* patterns were treated with Rietveld refinement to access lattice parameters and unit cell volumes (not shown) as indicators of structural changes. For better comparison to the later on discussed cubic (*Pm*3̄*m*) structure, the lattice parameters of the initially rhombohedral (*R3̄c*) crystalline structure were converted to pseudocubic values as done in ref. 26 or elsewhere.

**Fig. 4:**
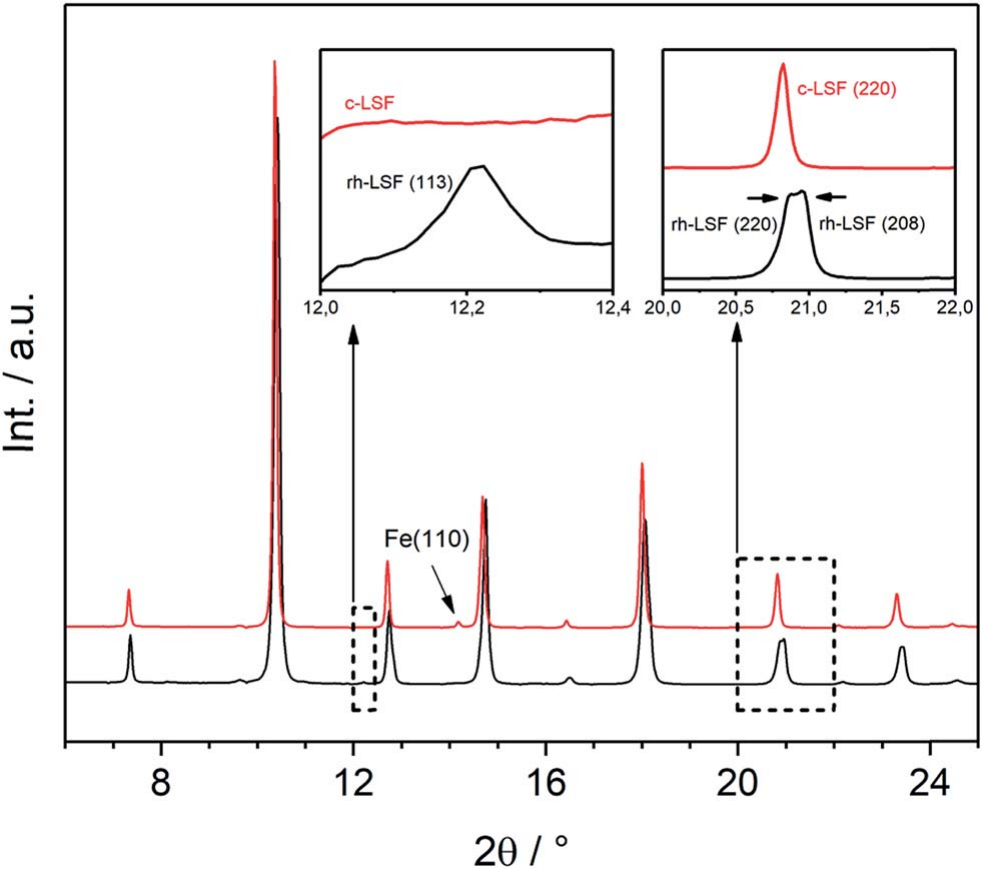
XRD patterns (*λ* = 0.4959 Å) of the initial rhombohedral LSF sample (black) and cubic LSF after cool-down to room temperature following the treatment in hydrogen to 873 K (red). The insets show the characteristic differences between the two different structures.

Both structures under consideration, the rhombohedral and the cubic, have mostly similar patterns in XRD. Nevertheless, they can be distinguished by certain features as demonstrated in Fig. 4, displaying the initial rhombohedral LSF sample (rendered as a black curve) and the specimen after treatment in hydrogen at 873 K (red), which is shown to exhibit a cubic structure. The rhombohedral structure exhibits an additional reflex at 2*θ* = 12.2° attributed to the (113) lattice plane, whereas the cubic structure has no reflex at this scattering angle (left inset). Moreover, some reflexes are split in the rhombohedral case, *e.g.* (220) and (208) at about 2*θ* = 21°, which cannot be resolved due to the close lattice spacings, whereas the cubic structure shows a single reflex at the same position. Additionally, in this case, the segregation of iron is visible by the Fe(110) reflex located at 2*θ* = 14°.

**Fig. 5:**
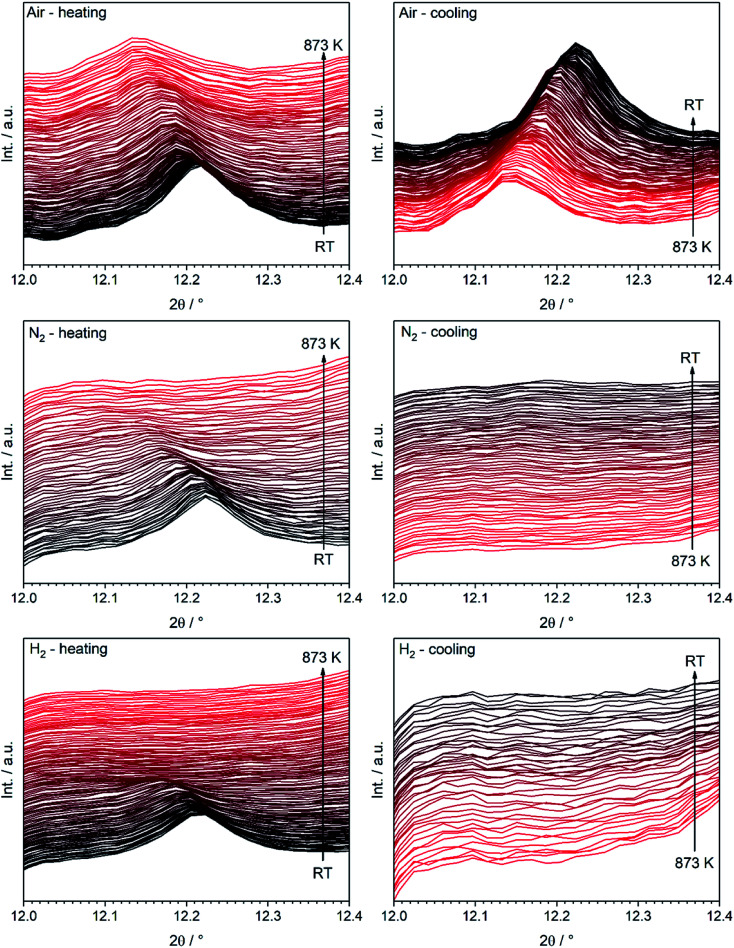
Temperature evolution of the (113) LSF reflex characteristic for the rhombohedral structure during treatment in all three gases between room temperature and 873 K (*λ* = 0.4959 Å). The color code is chosen as to show the first acquired diffractogram in black, with a continuous change to red as the temperature cycle progresses.

To clarify whether a structural transition takes place during heating/cooling in the three different atmospheres, the characteristic rhombohedral (113) reflex can be observed (Fig. 5). Whereas the reflex persists during heating in air (top left) as well as during subsequent cooling, it disappears when nitrogen or hydrogen atmospheres are applied (middle and bottom row): here, the (113) signal disappears during the heating step and does not re-appear upon cooling. The rhombohedral structure is thus shown to transform into the cubic structure and can be stabilized to room temperature if the heating is conducted in oxygen-deficient atmospheres. Our results are in agreement with those of Fossdal *et al.*,^16^ who observed the phase transformation also in oxygen-containing atmospheres upon heating to 1123 K and with those of Dann *et al.*^27^ reported earlier, who focused on the oxygen nonstoichiometry impact on the crystalline structure of La_1−*x*_Sr_*x*_FeO_3−*d*_ to reveal quantitative relationships between the concentration of vacancies and the structure of the oxide. In that case, the rhombohedral phase was also stabilized under oxidizing conditions.

**Fig. 6:**
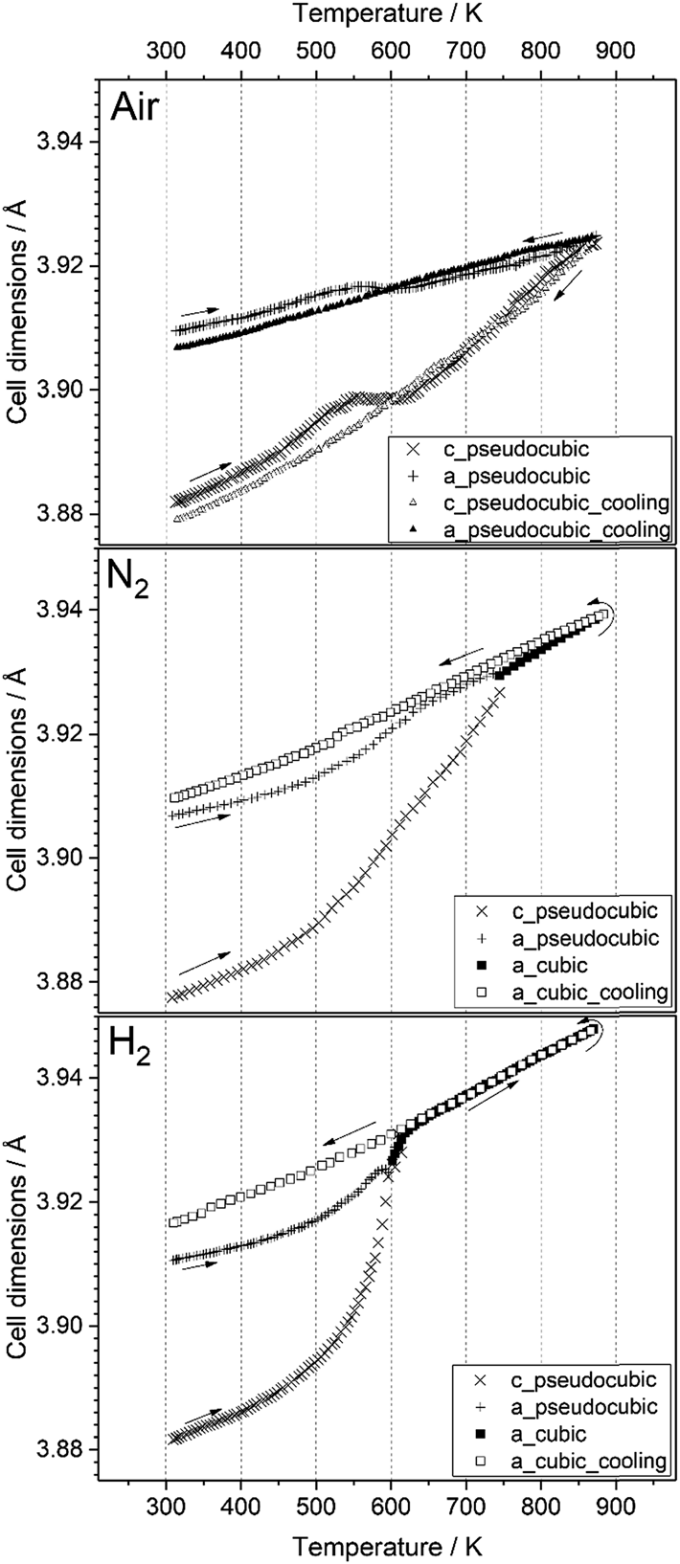
Temperature evolution of the unit cell parameters of LSF during treatment in air, nitrogen and hydrogen, up to 873 K. Arrows denote heating and cooling cycles, respectively.

We decided to analyze the (113) reflex instead of the peak splitting at 2*θ* = 21°, as the splitting or, more precisely, its disappearance at higher temperatures can be misleading: the (220) and (208) planes have different thermal expansion behavior, which results in an overlapping of the reflexes at elevated temperatures and an apparent “cubic” pattern at that 2*θ* region, which is not true in all cases as long as the (113) did not vanish. The structural changes can be followed as well, if the evolution of unit cell parameters during thermal treatments is investigated, as shown in Fig. 6. The unit cell parameters are given in pseudocubic (
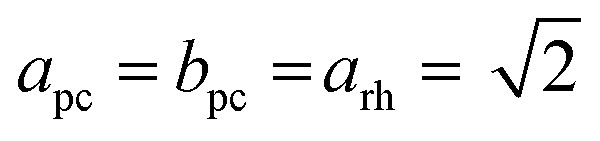
; 
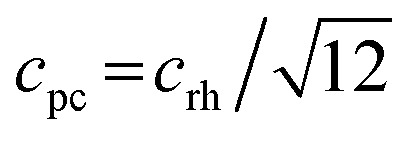
)^26^ or cubic values, depending on the actual crystal structure. For treatment in air, the rhombohedral perovskite (represented by the pseudocubic lattice parameters *a*_pc_ and *c*_pc_) exhibits thermal expansion until both values nearly intersect at the highest tested temperature of 873 K. Upon cooling, the lattice parameters relax back to slightly below the initial values, which, however, is still within the error of the refinement. Intersections of pseudocubic *a*_pc_ and *c*_pc_ values do take place in the case of nitrogen (at 750 K) and hydrogen (610 K) environments, where it can be attributed to a rhombohedral-to-cubic phase transformation. Until a maximum temperature of 873 K, the cubic lattice exhibits the expected linear thermal expansion. In all systems, the lattice parameters decrease linearly upon cooling down to room temperature, and do not suggest any further phase transformations.

As can be seen in Fig. 6, the temperature dependence of lattice parameter is generally not linear in the three atmospheres in the full temperature range. The lattice parameters of the sample heated in air increase with temperature up to 540 K, but then do not change, and further increase above 640 K. In contrast, both samples heated in N_2_ and H_2_ atmospheres display an increase in the lattice parameters with temperature in all temperature range of the experiments. Despite no phase transition is observed in the sample heated in air, its lattice parameters decrease steadily during cooling, but differ from those determined during heating in the temperature range 300–600 K. At high temperatures (*T* ≥ 600 K), the lattice parameters of the three samples during heating are very close to those calculated during cooling regardless the used atmosphere. In contrast, a rather big difference in the values of lattice parameters can be seen at lower temperatures. These results can be explained by the change in oxygen vacancy concentrations in the samples during the heating/cooling in different atmospheres.^28,29^ During heating, many oxygen vacancies are usually expected to be generated, while on cooling, the oxygen fill again these oxygen vacancies in the materials. The thermodynamic and kinetics of this process depend on the temperature as well as the treatment atmosphere. Thus, the lattice parameters of the sample heated in air are first increased with temperature because of the thermal expansion effect. In the temperature range 540–640 K, the diffusion of oxygen anions, which are generated by absorbing the oxygen from the atmosphere on the active sites of the material surface, becomes fast enough to diffuse through the crystal lattice, while the oxygen vacancies diffuse toward the surface.^28^ This process results in a decrease of the lattice parameters because the oxygen incorporation in the lattice will induce the valence increase of Fe ions as the charge compensation, resulting in an ionic radius decrease.^30^ In consequence, the decrease in lattice parameters due to oxygen incorporation compensates the increase due to the thermal expansion effect, thus, the lattice parameters of the sample heated in air stay relatively constant in this temperature range. In contrast, this constant tendency in the lattice parameters are not observed for the samples heated in N_2_ and H_2_ atmospheres because of the absence of oxygen. At higher temperatures (*T* ≥ 640 K), lattice oxygen release becomes predominant in the three atmospheres, which generates more oxygen vacancies and induce the valence decrease of Fe ions as the charge compensation. Therefore, the lattice parameters increase again due to the thermal and chemical expansion. The linear decrease in the lattice parameters of the three samples during cooling suggests that the oxygen contents do not change significantly and are very close to the equilibrium one in the cooled samples.

These results suggest that the rhombohedral-to-cubic transition upon heating LSF takes place only in oxygen-deficient conditions, *i.e.* in hydrogen as well as in nitrogen. If oxygen is supplied to the surface, as it is the case if the sample is heated in air, the transition does not take place, and the initial rhombohedral structure is preserved irrespective of the heat treatment.

From the *in situ* XRD data, it is obvious that an appropriately low oxygen partial pressure is needed to stabilize the cubic polymorph. The lack of oxygen in the gas phase is expected to cause oxygen vacancies to be formed, at first in the surface-near region and – at elevated temperatures with enhanced anion mobility – also in the deeper bulk regions. Enhanced reduction of the surface-near regions likely represents a process leading to the discussed Fe exsolution, which moreover requires a sufficient local Fe cation mobility. In general, the oxygen vacancies appear to be needed to stabilize the new phase at lower temperatures, very similar to *e.g.* the Zr–O system, where the tetragonal/cubic polymorphs of ZrO_2_ are stabilized by selected amounts of oxygen defects.^31^

**Fig. 7:**
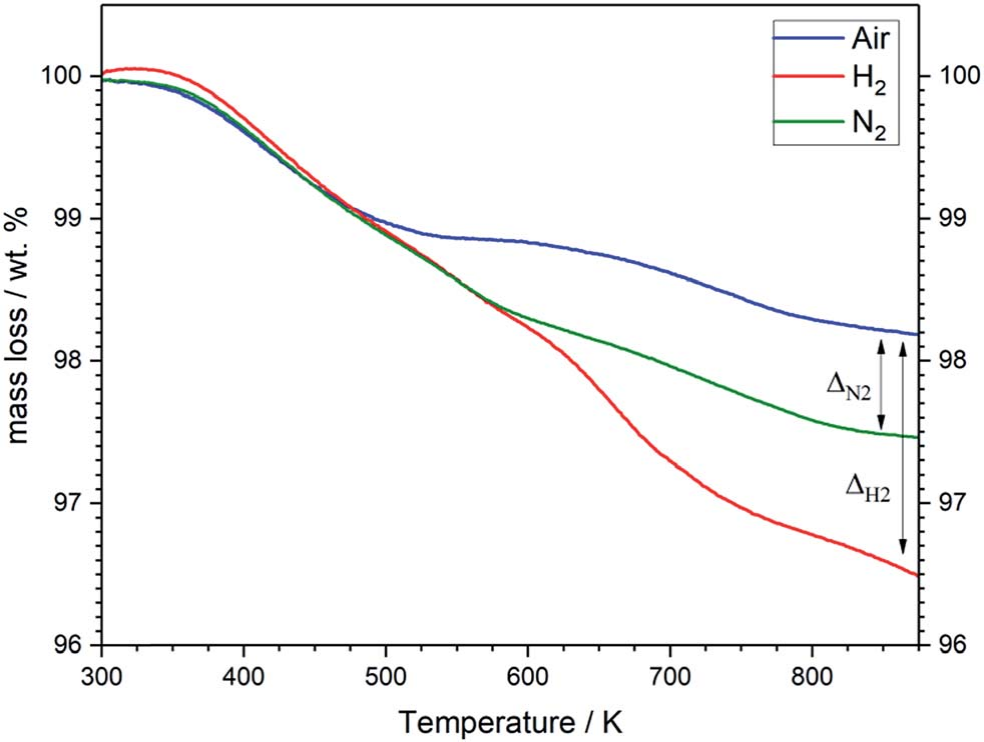
Thermogravimetric analysis of LSF samples treated in air (blue), H_2_ (red) and N_2_ (green) atmospheres. Heating rate: 10 K min^−1^.

The results of structural analysis are further supported by TG analysis (Fig. 7). The three specimens exhibit a weight loss of ∼1 wt% during heating from room temperature to about 500 K, which can be assigned to a desorption of physically absorbed water and gases. The weight of the sample heated in air remains almost constant in the temperature range 520–640 K before it starts to decrease again above 650 K. This result is in good agreement with the structural studies (Fig. 6), which can be attributed to the compensation of the loss in oxygen lattice by incorporating of oxygen anion absorbed on the surface from air atmosphere. In contrast, the specimens heated in N_2_ and H_2_ atmospheres show continuous decrease in the weight in this temperature range due to the absence of oxygen in the treatment atmospheres. At higher temperatures a significant weight loss is observed for all samples, which can be attributed the high release of oxygen lattice. The LSF specimen heated in H_2_ atmosphere undergoes larger total mass losses (≈3.5 wt%) in comparison to the samples heated in air (approximately 1.8 wt%). This indicates the generation of oxygen vacancies in the sample heated in H_2_ atmosphere. The mass loss in N_2_, 2.5 wt%, is about 0.7 wt% higher compared to air. The cubic (*Pm*3̄*m*) perovskite structure after treatment in hydrogen is most probably stabilized by defects, either by oxygen vacancies and/or Fe deficiencies generated during the heat treatment.

### 3.3. X-ray photoelectron spectroscopy

**Fig. 8:**
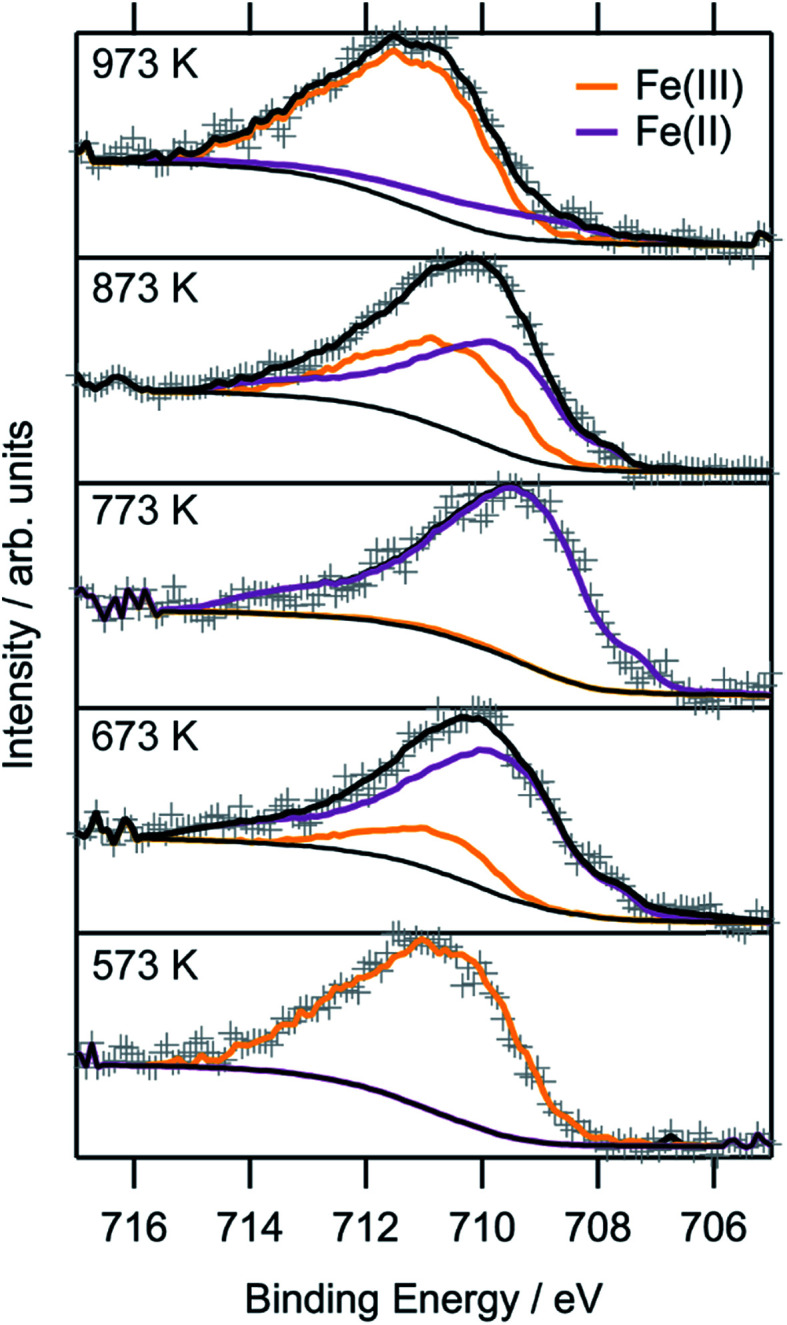
*Ex situ* Fe 2p XP spectra of the LSF samples after reduction at various temperatures between 573 K and 973 K for one hour each. The oxidation states were fitted using separately recorded spectra for FeO and Fe_2_O_3_, respectively.

**Fig. 9:**
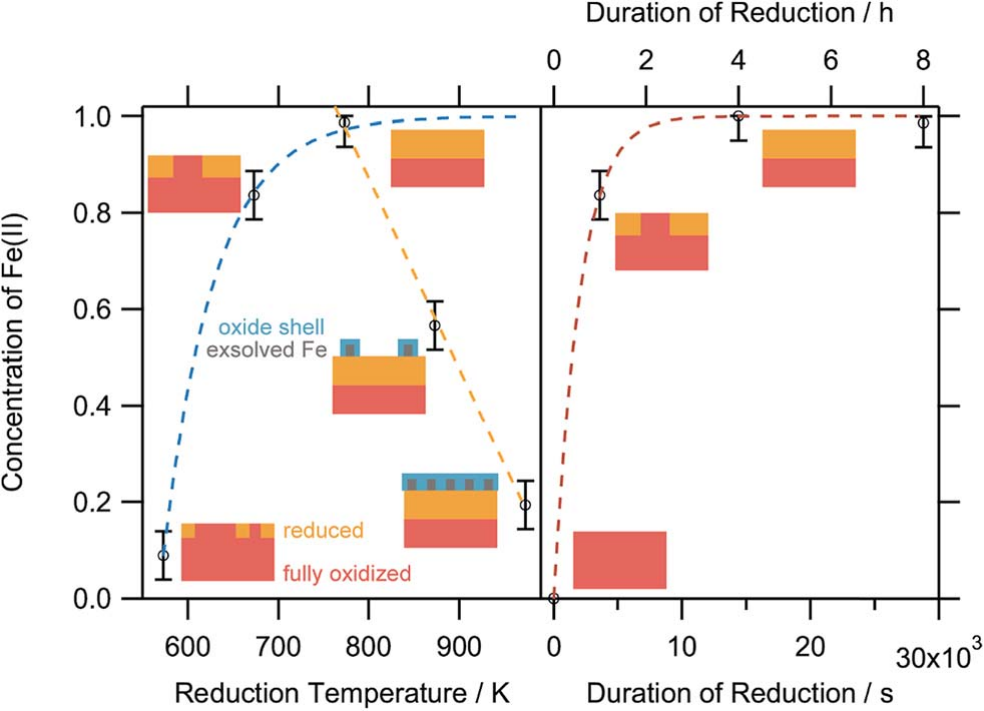
Relative concentrations of Fe^2+^ as a function of the reduction temperature (left panel) after treatment for 1 h each, and (right panel) the reduction time at 673 K. The small schemes serve as model perovskite surfaces to explain the respective oxidation state changes. Blue-dashed line: limited growth model. Orange-dashed line: linear temperature-dependence of Fe(ii). Red-dashed line: Avrami-type fit model for the isothermal increase of the Fe(ii) at 673 K reduction temperature.

To obtain a more thorough understanding of the presence of oxygen vacancies and reduced Fe species at least in the near-surface regions of the perovskite, XPS was employed. This technique allows the chemical analysis of the surface up to a depth of about 2–3 nm. According to the electrochemical potentials, Fe is more easily reduced than La or Sr: the standard reduction potential for the reduction of Sr^2+^ to Sr^0^ is −2.899 V with respect to the standard hydrogen electrode (SHE), whereas that for La^3+^ to La^0^ is also very negative at −2.379 V. The reduction of Fe^3+^ to Fe^2+^, on the other hand, is positive (*E*_0_(Fe^3+^|Fe^2+^) = 0.771 V). The reduction of Fe^2+^ to metallic Fe is more positive than the respective values for the other metals with *E*_0_ = −0.447 V.^32^ Hence, it is expected that the loss of lattice oxygen is compensated for by a change in the valence state of the Fe ions. The spectra of the Fe 2p region are depicted in Fig. 8. Only the 2p_3/2_ component is shown, as the important feature to distinguish Fe^2+^ and Fe^3+^ are the satellites at the higher BE side of the main peak. As the Fe 2p region is very complex, the oxidation states cannot be quantified by fitting simple Gaussian functions. However, quantification is possible using a weighted superposition of separately recorded spectra from the respective pure oxides. The spectrum of the sample reduced at 573 K already incorporates both oxidation states at 91 at% Fe^3+^ and 9 at% Fe^2+^. At a reduction temperature of 673 K, the satellite shifts further away from the main peak, creating a distinct shoulder at a BE of approximately 713 eV. In addition, the main peak exhibits a lower width compared to lower reduction temperatures. These changes in the spectrum arise from a large amount of Fe^2+^ species in the surveyed region: 84 at% of all iron ions are in the reduced state of Fe^2+^. A further rise of the temperature to 773 K reveals 99 at% Fe^2+^, which appears to not be oxidized during transport to the spectrometer for the *ex situ* measurement, due to its location within the parent LSF lattice. The Fe^2+^ satellite becomes less prominent after hydrogen treatment at 873 K as the concentration of Fe^2+^ is lowered again in favor of fully oxidized Fe^3+^, with the Fe^2+^ concentration being 57 at%. At 973 K, finally, the majority of all XPS-detectable Fe species (81 at%) is trivalent again. The evolution of the iron species is readily explained by the schematic representation in Fig. 9: Fe^2+^ increases up to 773 K is due to the redox reaction with hydrogen, as described above, resulting in the reduction of larger and larger fractions of the surface-near region of the perovskite. The apparent re-oxidation upon raising the reduction temperature can be understood on the basis of TEM analysis (Fig. 1): the exsolved iron particles are partially oxidized at the surface. Analogous behavior was reported for the Fe-rods in ref. 9, which feature a several-nanometer-thick oxide particle shell. Since the analysis depth of XPS for such oxides is about 2–3 nm, this renders the metallic core of these particles invisible to this technique. Thus, an increase in the Fe^3+^ content, which is accompanied by the respective decrease in Fe^2+^ (as no Fe^0^ is visible in the XP spectra) directly correlates to the amount of exsolved iron. It is, however, not straightforward to calculate the amount of metallic Fe as these particles sit on top of the perovskite and, thus, shadow the signal originating from the LSF grains, resulting in a lower detected amount of Fe^2+^. This would exaggerate the associated quantity of Fe^0^. However, a qualitative assessment of the exsolution process is still possible. The exsolution, thus, starts around 773 K and proceeds up to 873 K. At 973 K, where the Fe^3+^ concentration is largest again, it would appear that the surface exhibits a high density of Fe particles. However, careful analysis of the *ex situ* XRD data indicate that the stability limit of LSF has been exceeded, leading to beginning decomposition of LSF into (La,Sr)_3_Fe_2_O_7_.^33^ However, this transition would not explain the increased Fe^3+^ concentration on its own and, thus, the higher amount of Fe^3+^ can only originate from a larger number of exsolved and partially corroded Fe particles.

**Fig. 10:**
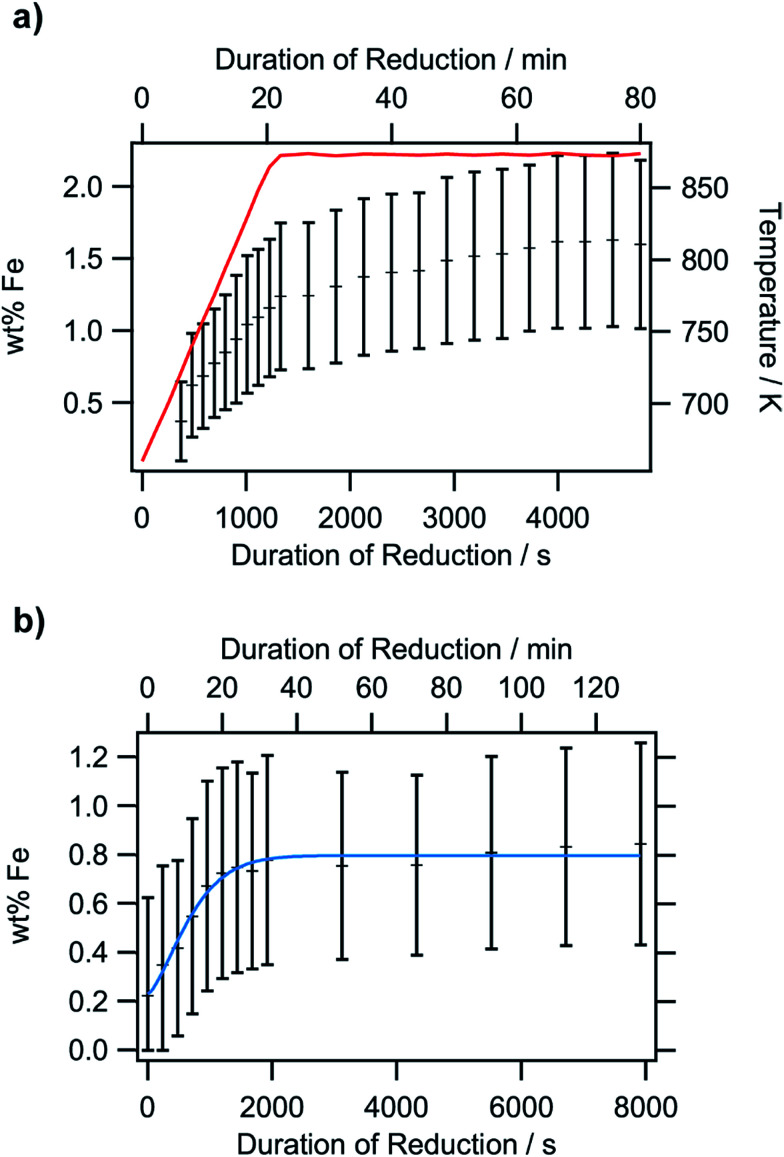
Rietveld analysis of the iron exsolution, based on isothermal *in situ* XRD experiments: temperature-dependent measurement in pure hydrogen from 673 K to 873 K and subsequent isothermal heating at 873 K (panel a) and an Ar/H_2_ mixture heated isothermally at 873 K (panel b). The crosses denote the Fe concentration and the red line in the upper panel the temperature. The blue line in the lower panel describes the Avrami fit applied to the data.

The XPS-derived Fe^2+^ percentage is plotted as a function of the temperature in the left panel of Fig. 9. The initial increase in the Fe^2+^ concentration due to the reduction of the perovskite can be described by a limited growth model (blue dashed curve) up to 773 K. However, those three points are not sufficient to make any detailed assertion with respect to the mechanism of the reduction process. Raising the temperature above 773 K leads to a linear decrease of the Fe^2+^ percentage with temperature (orange dashed line). Since this decrease of Fe^2+^ corresponds to an increase in the highest oxidation state of iron, Fe^3+^, it can be related to the amount of exsolved metallic iron. However, the linear decrease does not necessarily infer a linear temperature-dependence of Fe^0^—due to the fact that a passivated Fe particle will not only increase the amount of Fe^3+^ observed in the XP spectra, but also decrease the intensity of the Fe^2+^ peak (since the exsolved particle prevents the photoelectrons from the underlying perovskite from reaching the spectrometer). As this blocking of the perovskite surface is dependent on the particle size, it cannot easily be corrected for without further techniques such as *in situ* TEM. Also, bulk-sensitive techniques such as *in situ* XANES while measuring the sample current could yield the required information without being limited to the surface-near region. In the right panel of Fig. 9, the XPS results from an isothermal experiment are shown, where the reduction time at 673 K was varied. At this temperature, no crystalline iron exsolution was found for the treatment duration of 1 h. This graph further shows that the Fe^2+^ concentration does not drop upon heating for longer time periods (up to 8 h), indicating that no exsolution takes place at this temperature. This experiment allows an investigation of the reduction kinetics of the perovskite. The data can be fitted by an Avrami-type function (red dashed curve),*c*(Fe^2+^) = 1 − exp(−*k*^*n*^*t*^*n*^)with a reaction rate constant of approximately 5 × 10^−4^ s^−1^, and the exponent, *n*, being 1. A qualitative assessment reveals that the Fe^2+^ concentration shows the same behavior during the isothermal experiments as the temperature-varying ones at lower temperatures, with the Fe^2+^ concentration reaching saturation. To get a more detailed view on the kinetics of exsolution without the side effects present in the XPS data (*i.e.* the superposition of the Fe particles on the perovskite surface, thereby blocking the signal from the latter), Rietveld analysis of the intensity of the Fe(110) reflex in the *in situ* XRD diffractograms was conducted (Fig. 10). In Fig. 10 (panel b), the weight percentage of exsolved metallic iron for isothermal heating at 873 K in an Ar/H_2_ mixture is presented. Despite the large error bars leading to equally large errors for the fitting parameters, the kinetics clearly obey an Avrami-like behavior (fitting function shown as blue line). With start of the reaction set at 0 s in the plot, thus, disregarding the previous heating ramp for the fit, the rate constant is 0.0013 s^−1^, and the exponent is 1.45. The iron concentration reaches a maximum of approx. 0.8 wt% after about 1400 s and remains at that level. If the Ar/H_2_ mixture is replaced by pure H_2_, the plateau is reached much more quickly (Fig. 10, panel a). Fitting is not possible as the segregation of the iron particles already starts during the heating, rendering the changes during the isothermal period too small to yield valuable results. At 1.6 wt%, the largest concentration is twice that obtained for the Ar/H_2_ gas at the same temperature, and the change of the concentration during the isothermal period is slightly higher. Since the general shape of both plots resembles that of the Avrami function, the data suggest that the exsolution is also diffusion-controlled.^34^ This is understandable as the segregation of metallic iron depletes the surface-near regions of Fe. After this has finished, no further exsolution is possible until new iron is transported from the bulk to the surface. However, this also indicates that the exsolution itself, comprising the reduction, transport across the surface and crystallization of metallic iron, is quicker than the transport from the bulk. Here, isothermal periods at even higher temperatures would be required to discriminate whether the transport can become faster than the exsolution. This is, however, restricted by the stability limits of LSF.

**Fig. 11:**
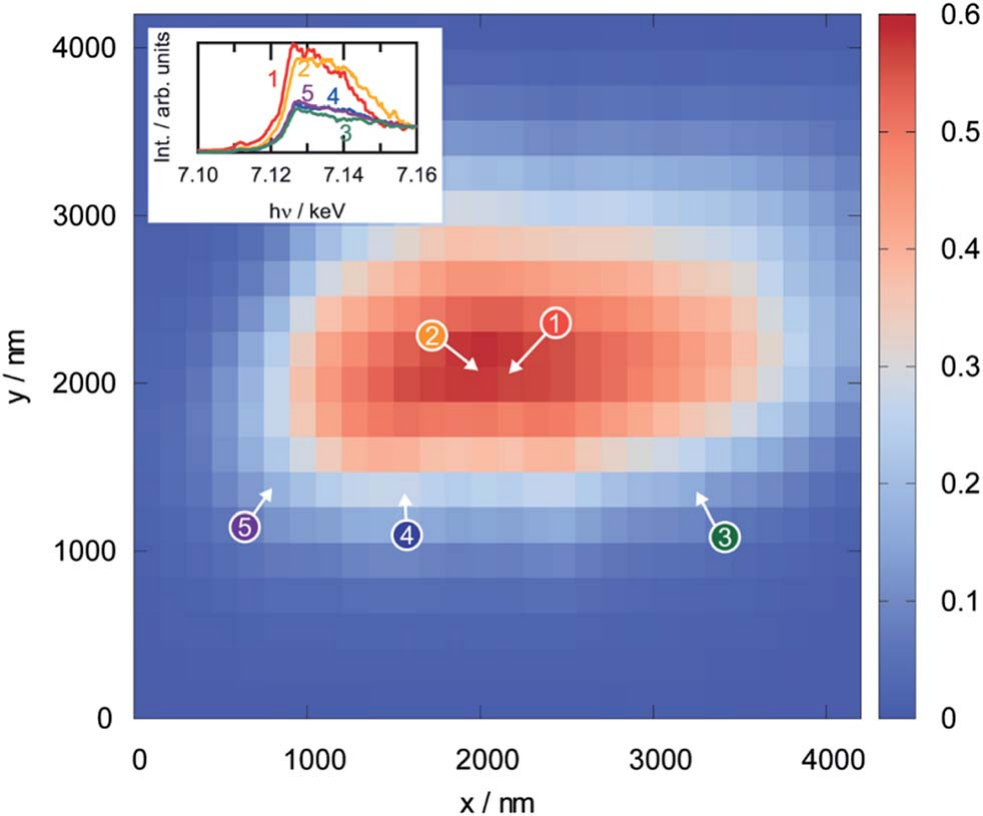
Thickness map of a single LSF particle reduced in pure hydrogen at 973 K for 1 h as obtained from scanning Fe Kα X-ray fluorescence mapping (large panel) and spatially-resolved Fe K-edge XANES spectra for the marked points (shown as insets). The latter can be separated into two distinct classes: bulk (1 and 2) and surface (3–5). The measurements were conducted in flowing He.

Since XPS is a surface-sensitive technique, it is not clear whether the reduction of the perovskite that was observed in the photoelectron spectra is limited to the region close to the surface, or whether it also affects the bulk. To assess this, scanning XANES was applied as an *ex situ* method (conducted in an inert He atmosphere after reduction) that is also sensitive to the electronic structure. Fig. 11 shows the results from the analysis of a perovskite particle that was reduced at 873 K prior to the measurement by displaying a thickness map calculated from the intensity in the X-ray fluorescence (XRF) spectra (obtained using a beam energy of 7.3 keV and a beam size of 1.57 × 0.96 μm and steps of 150 nm in *x*- and *y*-directions). The dots on the map show the positions where the Fe K-edge spectra, shown as insets, were recorded. These spectra can be grouped into two classes: the spectra recorded in the bulk region of the particle (numbers 1 and 2) look similar to each other, but distinctly different to those obtained at the surface regions (3 through 5). Although a density of states cannot be derived due to the high spectral noise, a combination of XANES and XPS infers that the reduced iron species in the perovskite are indeed restricted to the regions close to the surface, whereas the bulk still consists of Fe(iii).

## 4. Conclusions

Using a combination of various techniques, the iron exsolution in lanthanum strontium ferrite in reducing conditions was investigated. *In situ* XRD revealed that prior to the diffusion-controlled segregation/crystallization of the Fe particles, an irreversible phase transformation from the initial rhombohedral structure to the cubic polymorph takes place upon heating in hydrogen. This phase transition also occurs irreversibly in less reductive environment (*e.g.* nitrogen), whereas it is suppressed in air. This indicates that a low oxygen partial pressure is required to retain the structure after cool-down, with oxygen vacancies in the lattice stabilizing the cubic phase. To get an even more detailed insight into the exsolution, *in situ* XPS measurements are further required, allowing to follow the iron oxidation states without contact to air and, thus, to directly monitor the metallic iron as it is exsolved. As the morphology of the exsolved particles is significantly different with respect to the rod-like features discussed in previous work, further detailed studies with various gas environments exhibiting different reduction potentials to understand of the conditions required for the morphology change would also be required.

## Conflicts of interest

There are no conflicts of interest to declare.

## Supplementary Material

RA-008-C7RA12309D-s001
