# Supplementary material for: Structural investigations of La0.6Sr0.4FeO3−δ under reducing conditions: kinetic and thermodynamic limitations for phase transformations and iron exsolution phenomena
Source: RSC Adv. 2018 Jan 15;8(6):3120–31. doi: 10.1039/c7ra12309d (PMC9077552; doi:10.1039/c7ra12309d)
Supplement: RA-008-C7RA12309D-s001 [file RA-008-C7RA12309D-s001.pdf]

Electronic Supplementary Material (ESI) for RSC Advances.
This journal is © The Royal Society of Chemistry 2018

Supporting Information

Structural Investigations of $La_{0.6}Sr_{0.4}FeO_{3-\delta}$ under Reducing Conditions: Kinetic and Thermodynamic Limitations for Phase Transformations and Iron Exsolution Phenomena

Thomas Götsch[a,#], Lukas Schlicker[b,#], Maged F. Bekheet[b], Andrew Doran[c], Matthias Grünbacher[a], Corsin Praty[a], Mizuki Tada[d], Hirosuke Matsui[d], Nozomu Ishiguro[e], Aleksander Gurlo[b], Bernhard Klötzer[a], Simon Penner[a,*]

[a] *Institute of Physical Chemistry, University of Innsbruck, A-6020 Innsbruck, Austria*

[b] *Fachgebiet Keramische Werkstoffe, Institut für Werkstoffwissenschaften und -technologien, Technical University Berlin, 10623 Berlin, Germany*

[c] *Advanced Light Source, Lawrence Berkeley National Laboratory Berkeley, California 94720, USA*

[d] *Department of Chemistry, Graduate School of Science, Nagoya University, Furo-cho, Chikusa-ku, Nagoya 464-8601, Japan*

[e] *RIKEN SPring-8 Center, Sayo, Hyogo 679-5148, Japan*

# These authors contributed equally.

*Corresponding author: Simon Penner, simon.penner@uibk.ac.at, Tel: +43 512 507 58003

Keywords: hydrogen, oxygen defects, *in situ* X-ray diffraction, Rietveld analysis, perovskite

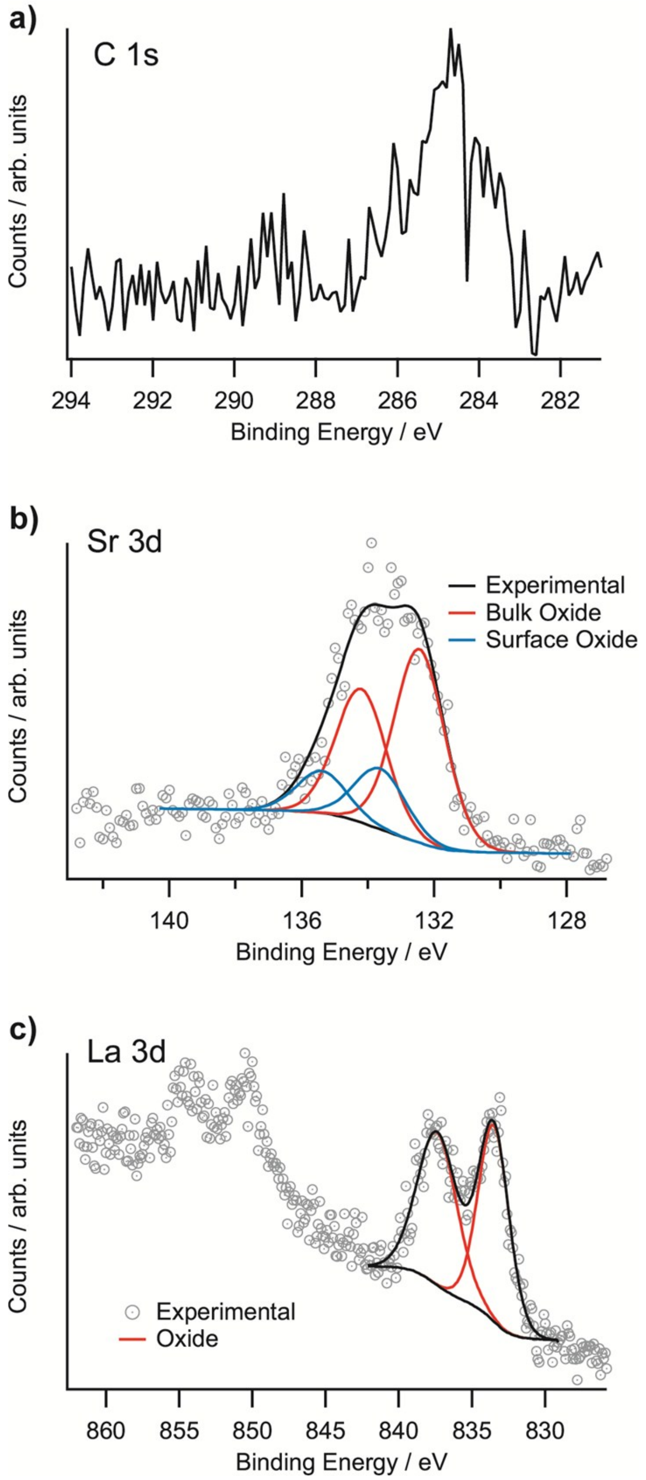

Figure S1: XP spectra of LSF – panels a), b) and c) show the individual C 1s, Sr 3d and La 3d peaks.
